# Supplementary material for: Insight into the Organization of the B10v3 Cucumber Genome by Integration of Biological and Bioinformatic Data
Source: Int J Mol Sci. 2023 Feb 16;24(4):4011. doi: 10.3390/ijms24044011 (PMC9961470; doi:10.3390/ijms24044011)
Supplement: Supplementary file 1 [file ijms-24-04011-s001.zip › S1_FINAL_SUMMARY_TABLE.html]

Contigs to chromosomes table


# Contigs to chromosomes table

#### **Description:**

This file allows the user to view the results of data integration for contig ordering in the B10 reference genome. The individual contig matches have been prepared in the form of interactive tables that allow easy filtering and sorting of the data. Detailed descriptions of the tables are provided below.

#### **Legend:**

- **Contig\_name** - The name of the contig in the B10 genome
- **Contig\_length** - The length of the contig in the B10 genome
- **Chromosome** - Chromosome according to markers used during B10v3 genome assembly
- **Strand** - The strand on which the chromosome is located according to markers used during B10v3 genome assembly
- **Chromosome\_9930** - Chromosome number found using RagTag program with use 9930 reference genome
- **Start\_9930** - The start of the contig in the genome after RagTag rearrangement with the use of 9930 reference genome
- **End\_9930** - The end of the contig in the genome after RagTag rearrangement with the use of 9930 reference genome
- **Strand\_9930** - The strand on which the chromosome is located in the genome after RagTag rearrangement with the use of 9930 reference genome
- **Chromosome\_Gy14** - Chromosome number found using RagTag program with the use of Gy14 reference genome
- **Start\_Gy14** - The start of the contig in the genome after RagTag rearrangement with the use of Gy14 reference genome
- **End\_Gy14** - The end of the contig in the genome after RagTag rearrangement with the use of Gy14 reference genome
- **Strand\_Gy14** - The strand on which the chromosome is located in the genome after RagTag rearrangement with the use of Gy14 reference genome
- **Chromosome\_blast\_9930db** - Blast result to the database created with the use of 9930 reference genome
- **Chromosome\_blast\_gy14db** - Blast result to the database created with the use of Gy14 reference genome
- **Chromosome\_according\_to\_DArT-seq\_analysis** - Assigned chromosome according to DArT-seq analysis results
- **STC\_sequence\_id** - STC sequence id in FISH analysis
- **Chromosome\_according\_to\_FISH\_analysis** - Assigned chromosome according to FISH analysis results
