# Supplementary material for: Insight into the Organization of the B10v3 Cucumber Genome by Integration of Biological and Bioinformatic Data
Source: Int J Mol Sci. 2023 Feb 16;24(4):4011. doi: 10.3390/ijms24044011 (PMC9961470; doi:10.3390/ijms24044011)
Supplement: Supplementary file 1 [file ijms-24-04011-s001.zip › S2_BLAST_results_9930db.html]

The result of a blast to the genome 9930


# The result of a blast to the genome 9930

**Description**:

The table below shows the results of blasting the longest peptide sequences of the B10 genome to the 9930 reference genome.

**Legend**:

**B10\_gene\_name** - Name of gene in B10 genome  
**B10\_contig** - Contig on which searched protein is located  
**gene\_start** - start of gene in B10 genome  
**gene\_end** - end of gene in B10 genome  
**sseqid** - Name of blasted gene in 9930 genome  
**pident** - percentage of identical matches  
**seqid** - name of contig on which gene is located in 9930 genome  
**start** - start of found gene in 9930 genome  
**end** - end of found gene in 9930 genome  
**strand** - strand on which gene is located in 9930 genome  
**length** - alignment length (sequence overlap)  
**mismatch** - number of mismatches  
**gapopen** - number of gap openings  
**qstart** - start of alignment in query  
**qend** - end of alignment in query  
**sstart** - start of alignment in subject  
**send** - end of alignment in subject  
**evalue** - expect value  
**bitscore** - bit score  
**annotation** - annotation of blasted gene  
**go** - gene ontology obtained from CuGenDBv2 database
