# Supplementary material for: Insight into the Organization of the B10v3 Cucumber Genome by Integration of Biological and Bioinformatic Data
Source: Int J Mol Sci. 2023 Feb 16;24(4):4011. doi: 10.3390/ijms24044011 (PMC9961470; doi:10.3390/ijms24044011)
Supplement: Supplementary file 1 [file ijms-24-04011-s001.zip › S4_BLAST_results_nr_3650.html]

The result of a blast to the nr database narrowed down to 3650 taxid.


# The result of a blast to the nr database narrowed down to 3650 taxid.

**Description**:

The table below shows the results of blasting the sequences of the longest peptides of the nr database narrowed down to 3650 taxid.

**Legend**:

**qseqid** - Name of query contig in B10 genome  
**sseqid** - Name of blasted gene in nr DB  
**pident** - percentage of identical matches  
**Annotation** - matched annotation using the Entrez database API  
**length** - alignment length (sequence overlap)  
**mismatch** - number of mismatches  
**gapopen** - number of gap openings  
**qstart** - start of alignment in query  
**qend** - end of alignment in query  
**sstart** - start of alignment in subject  
**send** - end of alignment in subject  
**evalue** - expect value  
**bitscore** - bit score
