# Supplementary material for: Insight into the Organization of the B10v3 Cucumber Genome by Integration of Biological and Bioinformatic Data
Source: Int J Mol Sci. 2023 Feb 16;24(4):4011. doi: 10.3390/ijms24044011 (PMC9961470; doi:10.3390/ijms24044011)
Supplement: Supplementary file 1 [file ijms-24-04011-s001.zip › S7_genes_to_contigs.html]

Contigs to genes table


# Contigs to genes table

#### **Description:**

The table below shows the assignment of genes to contigs in the B10 genome. The order of genes in the list corresponds to the order of positioning on each contig.

**Contig\_name** - the name of contig in B10 genome  
**Genes** - genes which are located on contig in B10 genome
