# Supplementary material for: Insight into the Organization of the B10v3 Cucumber Genome by Integration of Biological and Bioinformatic Data
Source: Int J Mol Sci. 2023 Feb 16;24(4):4011. doi: 10.3390/ijms24044011 (PMC9961470; doi:10.3390/ijms24044011)
Supplement: Supplementary file 1 [file ijms-24-04011-s001.zip › Tables S1-S7.pdf]

Table S1: Final report with information on the assignment of contigs to chromosomes and the integrated results of the respective analyses.

Table S2: BLAST results of the search of the 9930 database.

Table S3: BLAST results of the search of the Gy14 database.

Table S4: BLAST results of the search NR database narrowed down to 3650 taxon.

Table S5: Blast results of DArT-seq data.

Table S6: Blast results of the search NR database.

Table S7: Table describing the assignment of genes to contigs in the B10v3 genome.
